# Supplementary material for: Integrating status-neutral and targeted HIV testing in Zimbabwe: A complementary strategy
Source: PLoS One. 2025 Feb 24;20(2):e0302803. doi: 10.1371/journal.pone.0302803 (PMC11849839; doi:10.1371/journal.pone.0302803)
Supplement: Attachment — Submitted filename: Comment to the authors.docx [file pone.0302803.s002.docx]

Manuscript title: A Status-Neutral Approach to HIV – Is Targeted Testing Still Relevant South of

Sahara?

Manuscript ID: PONE-D-24-14652

Authors: Hamufare Dumisani Dumisani Mugauri, Ph.D. Public Health

O Mugurungi, MD, MPH

Joconiah Chirenda, MBChB, MPH, PhD

Kudakwashe Takarinda, Bsc Maths & Statistics, Msc Biostatistics, PhD

Mufuta Tshimanga, MD, MPH

Prosper Mangwiro, Bsc Statistics, MPH

Dear authors thank you for coming up with good title and good work. I reviewed the article and have some comments.

**Title**: please specific it, since it confuses the reader, south of sahara while the study in one country?

**Abstract**:

1. In method add the final sample size included
2. Conclusion should be conclusion but not comparison, revise the conclusion

**Introduction**: The introduction is interesting but the previous literatures and practice of status neutral in Africa not well addressed

Method and materials

Study design: say only cross sectional

1. Did you only include 1 October 2023 to 31 December 2023 tested data, what about those tested in the community?what about those tested in non-government organization?
2. Introduction Argue the reader what is new on Status-Neutral Approach to HIV over PICT, VCT,index testing, **Social Network-Based HIV Testing and others strategies**
3. Did you included the minors?
4. As we know DHIS2 is not accessed by other third part since it accessed by government organization, how did you access it?
5. Our study was exempted from ethical clearance, how since HIV is sensitive issue and DHS2 isnot open access, I think this research had ethical problem?
6. The result seems report please rewrite it
7. Discussion please remove the limitation and strength on this section and write after is before conclusion
8. Discussion should be discussion, remove the results in discussion and discuss with previous literatures
9. In clonclusion what overway status-neutral testing to other tasting strategy, Is more effective than PICT, VCT,index testing, **Social Network-Based HIV Testing and others strategies**
