# Supplementary material for: Integrating status-neutral and targeted HIV testing in Zimbabwe: A complementary strategy
Source: PLoS One. 2025 Feb 24;20(2):e0302803. doi: 10.1371/journal.pone.0302803 (PMC11849839; doi:10.1371/journal.pone.0302803)
Supplement: Attachment — Submitted filename: peer review and feedback_Joshi.docx [file pone.0302803.s004.docx]

**peer review and feedback for the document, “A Status-Neutral Approach to HIV – Is Targeted Testing Still Relevant in Zimbabwe?”**

**Reviewed by – Dr. Prachi Joshi.**

**General Overview**

The manuscript addresses a critical and contemporary public health topic: the integration of status-neutral testing with targeted testing in Zimbabwe. The study is comprehensive and methodologically sound, offering data-driven insights into HIV testing strategies. However, there are areas where clarity, structural consistency, and emphasis on the research question can be improved.

**Strengths**

1. **Relevance**: The topic is highly relevant, addressing an innovative HIV testing paradigm in a resource-constrained, high-burden setting.

2. **Methodology**: The use of cross-sectional data from multiple provinces ensures generalizability within Zimbabwe.

3. **Data Analysis**: The statistical approach is robust, including multivariate analyses that address confounding variables.

4. **Comprehensiveness**: The authors balance quantitative findings with policy and practice implications.

**Areas for Improvement**

**1. Title and Abstract**

• **Clarity**: The title implies a dichotomy (“is targeted testing still relevant?”) while the study shows complementarity. Consider a more reflective title, such as:

*“Integrating Status-Neutral and Targeted HIV Testing in Zimbabwe: A Complementary Strategy”*

• **Abstract**: The abstract includes too much detail on statistical findings, which could be overwhelming. Instead, highlight the key takeaway: status-neutral and targeted testing are complementary.

**2. Introduction**

• The introduction does not adequately address the novelty of status-neutral approaches globally and regionally. Expand on its application outside Zimbabwe to contextualize its relevance.

• Clarify why Zimbabwe serves as a unique case study compared to other sub-Saharan countries.

• Strengthen the argument for combining targeted and status-neutral strategies as complementary.

**3. Methods**

• **Sample Size Calculation**: Although a robust approach is mentioned, its relevance to the presented data could be clearer. For example, there is a mismatch between the calculated sample size and the reported 23,058 patients.

• **Ethics Section**: While de-identified data are exempt from clearance, sensitivity around HIV data warrants a detailed explanation of privacy safeguards.

**4. Results**

• The results section reads like a report rather than a scientific narrative. Focus on linking findings to the study’s primary aim (complementarity of the two testing strategies).

• Highlight specific examples of how the status-neutral approach improved linkage to prevention and treatment.

• Consolidate the discussion of demographic disparities (e.g., male testing rates and positivity) into actionable insights.

**5. Discussion**

• The discussion reiterates results without sufficiently engaging with existing literature. Integrate comparative findings from sub-Saharan Africa to strengthen the global relevance of your conclusions.

• Clarify the mechanisms by which status-neutral testing complements targeted testing beyond mere statistics (e.g., behavioral factors, system efficiencies).

• Move limitations and strengths to a dedicated section, as per journal conventions.

**6. Figures and Tables**

• Figure legends and table captions should be more descriptive, emphasizing the findings they illustrate.

• For example, Figure 2 could include a brief note on how linkages improved due to the combined strategy.

**7. Language and Grammar**

• Minor grammatical issues exist throughout. For instance, simplify phrases like:

*“Targeted testing and status-neutral testing are related concepts which are complimentary.”*

to

*“Targeted and status-neutral testing are complementary strategies.”*

• Ensure consistent terminology (e.g., “positivity ratio” vs. “positivity rate”).

**8. Policy Implications**

• While the manuscript briefly addresses policy, expand this section with clear recommendations. How should Zimbabwe and similar countries allocate resources between targeted and status-neutral testing?

**Specific Recommendations**

1. Revise the conclusion to clearly state how findings align with the study’s objectives. Avoid introducing new information.

2. Address potential biases (e.g., reliance on DHIS2 data) and how they were mitigated.

3. Strengthen the narrative flow between sections—particularly the connection between methods, results, and discussion.

This manuscript provides valuable insights but requires refinement to align its presentation with its scientific and practical contributions. By addressing these recommendations, the authors can enhance the manuscript’s clarity, impact, and readiness for publication.
