# Supplementary material for: Integrating status-neutral and targeted HIV testing in Zimbabwe: A complementary strategy
Source: PLoS One. 2025 Feb 24;20(2):e0302803. doi: 10.1371/journal.pone.0302803 (PMC11849839; doi:10.1371/journal.pone.0302803)
Supplement: Attachment — Submitted filename: PONED-24-14652_R1.docx [file pone.0302803.s005.docx]

| **Primary Outcome of interest** | Lack of conceptual and operation definition was provided.  180-181: The primary outcome of interest is the positivity to HIV.  Based on the data given and analysis, effectiveness of the status-neutral approach in targeted HIV testing can not be performed since no intervention was given and data was used was secondary only. |
| --- | --- |
| **Analysis and statistics** | 209: Please include the significant OR in Table 3 for the narrative. Also, please explain indicate the variables. Was the risk screen before testing and testing district not in Table 2 not included in the adjusted model?  Why was the over variables not included? like HIV status etc?  210: Assessment of risk profile used was log-binomial regression but in Table 3 variables were analyzed using Poisson regression. can you explain?  In addition, since your design is cross-sectional relative risk may not be applicable but prevalence ODDs ratio maybe use.  211-213: If your decision was based on the log-binomial regression modelling then you should specific what variables were selected or added in the adjusted models. |
| **Discussion** | 251-252 Not enough data to support that HIV testing complements targeted HIV testing.  257-263 – should be part of the results  264-270 – How is 75% over all positivity in screened patients suggest importance of self-screening options to those at risk? Discussion of the algorithm should support this.  271-274 – same with 264-270.  275-279 – your statement might be bias since you did not present data of negative individuals such as their status.  280- what was the data that supports importance of status-neutral approach? it was not operationally defined.  372-312. Discussion of Table 3 or associated variables was lacking. |
| **Conclusion** | 338-339 this negates your statement about status-neutral approach.  Conclusion should be based on the results and discussion. |
| **Other comments** | - Selection and information bias as evident in the data presented particularly misclassification of subject’s exposure due to limited data extracted. - There should be a clear inclusion and exclusion criteria for the selection of data. - Author discussion and conclusion about the algorithm was not supported by the results and justified. |
