# Supplementary material for: Integrating status-neutral and targeted HIV testing in Zimbabwe: A complementary strategy
Source: PLoS One. 2025 Feb 24;20(2):e0302803. doi: 10.1371/journal.pone.0302803 (PMC11849839; doi:10.1371/journal.pone.0302803)
Supplement: Attachment — Submitted filename: A status neutral approach to HIV_review_KM.docx [file pone.0302803.s006.docx]

**A Status-Neutral Approach to HIV – Is Targeted Testing Still Relevant in Zimbabwe?**

Thank you for the opportunity to review this paper highlighting the benefits of employing a combined status neutral and targeted HIV testing approach in low resource settings. The study found that status neutral approach and targeted testing approaches complement each other and can help prevent HIV transmission, promote linkage to care and treatment services, and the strategic use of limited resources to end the HIV epidemic. This work is an important contribution to the field and should be considered for publication with some revisions. Below I provide a few comments for the authors to consider.

**Introduction**

1) The sentences from lines 99-102 are unclear. Lines 99-100 is an incomplete sentence and lines 101-102 the concept of variability is not explained, I encourage the authors to revisit this paragraph for clarity.
2) In lines 103-105, it would be useful for readers if the authors could provide a brief overview of how the status neutral approach has been integrated into existing HIV testing frameworks in sub-Saharan Africa.
3) Lines 107-108 outline the knowledge gap the authors intend to fill, this is also a good opportunity for the authors to expand upon the reason why it is important to examine this dual strategy and better position their work and where it fits/its potential contribution/use.
4) Last paragraph, lines 124-127, the aims of the paper could be better outlined. Authors should emphasize the examination of the complementariness of status neutral and targeted approach to HIV testing.

**Methods**

1) This section could be structured a bit better to help with the flow. For example, in lines 130-131 under the authors just list “cross-sectional”. I encourage the authors to get rid of the study design heading and instead write a descriptive sentence that includes the study design but also adds some context about the study, including discussion of the data source, etc. and other important aspects that readers should be aware of.
2) Authors should consider removing the sub-headings in line 133 and 138.
3) In line 142 authors mention four subunits of ATP but name only three.
4) Description of study sites, lines 154-165, at times is unclear, authors should revise to improve readability. Also, the discussion of each province is unbalanced. I would also suggest the authors include data on the HIV prevalence and incidence rates in the four provinces. The authors discuss how these provinces were selected later on in the study, but that information should be moved to here to enhance comprehension.
5) Discussion of the sampling strategy should precede the discussion of the client population; authors should revise the order in which the sampling strategy and sample are discussed to improve comprehension.
6) In line 201 the authors states that no personal identifiers were collected but in line 199 it states that “name of patient” was collected from the report.
7) In line 217-219 authors explanation for why the study was exempt from ethical clearance makes it seem like DHIS2 database does not collect personally identifiable information, authors should revise justification and state that they received de-identified data if that was the case, and briefly explain the process.
8) It would be helpful for the authors to briefly describe the complementary approach that fuses status neutral and targeted testing, is there a model that can be referenced? Both approaches are described but there is not a clear description of the kind of testing the authors seem to be advocating for.
9) Authors should describe the variables, including what screening for an HIV test, linkage to post-test services, and specific services entail.

**Discussion**

1) The authors discuss their results and implications very succinctly. It would be helpful if the authors provided some discussion on specifically how this approach can be implemented and why, to illustrate the contribution of the findings of study and/or discussion of how the authors see their findings directly affecting health care service delivery.
2) Authors do not offer actionable steps or address policies or specific things that need to change and/or stakeholders that need to be involved to enhance the delivery of the status neutral-targeted HIV testing approach in Zimbabwe and beyond. Description of barriers to its implementation is also warranted in this vein.
3) Authors described interesting findings and based on this should consider including recommendations for future research.

**Minor points**

1) Sentence structure, ensuring proper spacing and punctuation surrounding citations e.g. period comes after the citation not before, etc.
2) Some parts could be written more clearly, I encourage the authors to revise the language to improve flow, readability and enhance comprehension. The authors could also consider seeking support from a copy editor.
