# Supplementary material for: Integrating status-neutral and targeted HIV testing in Zimbabwe: A complementary strategy
Source: PLoS One. 2025 Feb 24;20(2):e0302803. doi: 10.1371/journal.pone.0302803 (PMC11849839; doi:10.1371/journal.pone.0302803)
Supplement: Attachment — Submitted filename: A status neutral approach to HIV_2nd review_KM.docx [file pone.0302803.s008.docx]

**A Status-Neutral Approach to HIV – Is Targeted Testing Still Relevant in Zimbabwe?**

Thank you for providing the opportunity to re-review the paper post revisions from the authors. I am satisfied with all the changes. One minor suggestion I have is that, in addition to the detail provided on the included provinces, I recommend that the authors to consider including the prevalence rates for each province into the manuscript.
