# Supplementary material for: Integrating status-neutral and targeted HIV testing in Zimbabwe: A complementary strategy
Source: PLoS One. 2025 Feb 24;20(2):e0302803. doi: 10.1371/journal.pone.0302803 (PMC11849839; doi:10.1371/journal.pone.0302803)
Supplement: Attachment — Submitted filename: Mugauri_Detailed responses to reviewer comments.doc [file pone.0302803.s003.doc]

**Point-by-Point Response to Editor and Reviewers of Paper PONE-D-24-14652 :**

We thank you all for your time and dedication in going through this paper and we acknowledge the reviews and comments from the editor and reviewers. We provide a point-by-point response to each comment with each response introduced by the words “AUTHOR RESPONSE” in capital letters. We have revised the paper accordingly in response to these comments as indicated in our responses below and in tracked changes in the manuscript. We hope that the revised paper meets your expectations and may now be suitable for publication in your journal.

Yours sincerely,

Hamufare Mugauri

**Point by Point Response to reviewer comments**

1. REVIEWER COMMENT
   Dear authors thank you for coming up with good title and good work. I reviewed the article and have some comments.

**Title**: please specific it, since it confuses the reader, south of sahara while the study in one country?.
AUTHOR RESPONSE

Thank you for your appreciation and the comment. We have revised the title to be specific to Zimbabwe.

2. REVIEWER COMMENT**:** **Abstract**:
   a. In method add the final sample size included
   b. Conclusion should be conclusion but not comparison, revise the conclusion

AUTHOR RESPONSE: Thank you for pointing this out. We have revised the entire abstract to ensure that the conclusions are well aligned. We included the entire population of documented tests for the period studied and did not calculate a sample.

3. REVIEWER COMMENT**:** **Introduction**: The introduction is interesting but the previous literatures and practice of status neutral in Africa not well-addressed

AUTHOR RESPONSE: Thank you for this comment. Considering that Status Neutral is a new concept, there is paucity of data on its implementation in Africa. However, we managed to add a paragraph that speaks to the concept in relation to the concept as follows: “In Africa, particularly in sub-Saharan regions, the status-neutral approach has been integrated into existing HIV testing frameworks to enhance the effectiveness of HIV prevention and treatment programs. Countries like Zimbabwe have been at the forefront of implementing this approach alongside targeted testing methods [7]. This dual strategy is yet to be fully understood, highlighting a knowledge gap on how it relates to targeted testing concept”

4. REVIEWER COMMENT: . Study design: say only cross-sectional

AUTHOR RESPONSE: Thank you, this has been corrected as suggested.

5. REVIEWER COMMENT**:** Did you only include 1 October 2023 to 31 December 2023 tested data, what about those tested in the community?what about those tested in non-government organisations?

AUTHOR RESPONSE: Thank you for this question. The data we included is for 1 October 2023 to 31 December 2023 only and included all testing data from the community and partners. This data is all entered into DHIS2, a national electronic database.

6. REVIEWER COMMENT**:** Introduction Argue with the reader what is new on the Status-Neutral Approach to HIV over PICT, VCT,index testing, **Social Network-Based HIV Testing and others strategies**

AUTHOR RESPONSE: Thank you for the concept. The status neutral concept is a new concept itself as introduced in the introduction as follows “Status-neutral HIV testing is a novel approach to HIV education, testing and treatment that accentuates a continuum of care regardless of the individual’s HIV diagnosis….”.

7. REVIEWER COMMENT: Did you include the minors?

AUTHOR RESPONSE: This study only included 15 year olds and above, who are eligible to consent to HIV Testing in Zimbabwe. Line 165-170 reads “*All clients who were tested for HIV and documented in HIV Testing registers, at the 36 sampled facilities between* *1 October 2023 to 31 December 2023 and aged 15 years and above, were included in the study*”

8. REVIEWER COMMENT**:** As we know DHIS2 is not accessed by other third part since it accessed by government organization, how did you access it?

AUTHOR RESPONSE: Thank you for this question. The lead investigator is attached to the Ministry of Health, AIDS AND tb Programme at Head office and has access rights to DHIS2, Further, another author, Dr Owen Mugurungi is the Director AIDS & TB Programe and has access rights to DHIS2 too.

9. REVIEWER COMMENT**:** Our study was exempted from ethical clearance, how since HIV is sensitive issue and DHS2 isnot open access, I think this research had ethical problem?.

AUTHOR RESPONSE: Thank you for raising this important concern. The study in question was exempted from ethical clearance based on specific criteria outlined by the institutional review board (IRB). While HIV is indeed a sensitive issue, the exemption was granted because the study utilized de-identified data from the DHIS2 system, ensuring that no personal identifiers were accessible. This approach aligns with ethical guidelines that prioritize participant confidentiality and data protection. Moreover, the study adhered to the principles of ethical research by ensuring that all data used were anonymized and aggregated, thus minimizing any potential risks to individuals. The exemption was also based on the fact that the research did not involve direct interaction with human subjects, which typically necessitates a more rigorous ethical review process

10. REVIEWER COMMENT: The result seems report please rewrite it

AUTHOR RESPONSE: Thank you for your feedback. I understand that the results section may come across as more of a report rather than a detailed analysis. We have revised this section to ensure it provides a clearer interpretation of the data, highlighting key findings and their implications.

11. REVIEWER COMMENT: Discussion please remove the limitation and strength on this section and write after is before conclusion

AUTHOR RESPONSE: Thank you for your suggestion. We have removed the limitations and strengths from the Discussion section and place them in a new section titled “Limitations and Strengths,” which is positioned just before the Conclusion. This adjustment will help streamline the Discussion section and provide a clearer structure to the manuscript..

12. REVIEWER COMMENT: Discussion should be discussion, remove the results in discussion and discuss with previous literatures

AUTHOR RESPONSE: We acknowledge your opinion. We have revised the entire discussion section to be more scientifically correct and relate our discussion points to previous literature

13. REVIEWER COMMENT**:** * Line 170 reads “No patient level data was collected”, I presume here authors mean no “personal identifiers” (to ensure privacy and confidentiality) because age, sex… are still patient level data.

AUTHOR RESPONSE: Thank you for pointing this out. We have revised to read: “*No personal identifiers were collected.”*

14. REVIEWER COMMENT: The last paragraph of the discussion should be for limitations of the study. I don’t think study strengths add any value to the discussion. In addition to the limitations presented, authors must discuss their implications on the results discussed as well as the good side to it worth noting.

AUTHOR RESPONSE: Thank you for pointing this out. The entire Discussion has been revised and the comment addressed.

15. VREVIEWER COMMENT: The discussion and conclusion sections stem from the methods and results sections so it becomes very difficult to discuss something that has not be succinctly presented. Authors now discuss status-neutral and targeted approaches, but readers have not read much that helped them to understand the role that the first played in the second. There is a disconnection between the introduction and discussion sections to the methods and results sections

AUTHOR RESPONSE: Thank you for your valuable feedback. We understand the importance of ensuring that the discussion and conclusion sections are clearly connected to the methods and results sections. We have revised these sections to provide a more succinct and coherent presentation of our findings.

16. REVIEWER COMMENT**:** In my opinion the title of this paper doesn’t correlate with the aim of this study. To answer the question on the relevance of targeted testing, the article needs to showcase the contribution of targeted testing to positivity ratios and linkage to post-test services thus provide elements to justify why it should either be stripped off or maintained. Lines 34-36 rather present study aim to analyze the role of status-neutral approach in the effectiveness of targeted testing. This means thus that the notion of complementarity is already being promoted by the study aim (as backed by the conclusion of the abstract), why then leave the impression of mutual exclusiveness?

AUTHOR RESPONSE: Thank you for your insightful comments. We appreciate your feedback and have carefully considered your suggestions.

1. **Title Correlation with Study Aim:**
   - We acknowledge your concern regarding the correlation between the title and the study aim. To address this, we have revised the title to be more specif to Zimbabwe than saying South of sahara. However, we prefer that it remain a rhetoric question that it is, but addressing all the concerns you raised: *“****A Status-Neutral Approach to HIV – Is Targeted Testing Still Relevant in Zimbabwe?****.”*
2. **Showcasing Contribution of Targeted Testing:**
   - We agree that it is crucial to highlight the contribution of targeted testing to positivity ratios and linkage to post-test services. We have added a section in the results and discussion that specifically addresses these aspects. This includes data on positivity ratios and detailed analysis of how targeted testing contributes to effective linkage to post-test services. These additions are within the entirely revised results and discussions sections
3. **Clarifying Complementarity vs. Mutual Exclusiveness:**
   - We understand the importance of clarifying the notion of complementarity as opposed to mutual exclusiveness. We have revised the relevant sections (lines 34-36 and the conclusion of the abstract) to emphasize that the status-neutral approach complements targeted testing rather than being mutually exclusive.
17. REVIEWER COMMENT**:** Lines 47-48 read as thus “…from 66% (n=15,289) of the total tests conducted” which is confusing given authors earlier mentioned that 23,058 HIV tests were done. Authors have to clarify such that a reader will easily understand the difference between “HIV tests done” and “total tests conducted

AUTHOR RESPONSE: Thank you for your valuable feedback. We understand the confusion and have made the following clarifications to ensure the distinction between “HIV tests done” and “total tests conducted” is clear: We have revised the manuscript to clearly added that total tests done are actually total **HIV** tests done.

18. REVIEWER COMMENT: Lines 48-51, authors use a considerate portion of the results subsection of the abstract to present factors associated to HIV seropositivity (test positive or test HIV positive) as one of the key findings which brings me to this question. How does these help in answering the question on the role of status-neutral approach on targeted testing? Its ok to put some unexpected findings but priority must be given to elements that seek to answer the research question and that question here seeks to show the role of status-neutral approach to effectiveness of targeted testing (based on the study aim) not identify factors associated with HIV seropositivity. This comment is valid for the main text (analysis, results and discussion sections) of the paper too.

AUTHOR RESPONSE**:** Thank you for your insightful comments. We appreciate your feedback and have carefully considered your suggestions.

1. **Relevance to Research Question:**
   - We acknowledge that the primary aim of our study is to evaluate the role of the status-neutral approach in the effectiveness of targeted testing. We agree that the focus should be on elements that directly address this research question. To this end, we have revised the abstract and main text to prioritize findings that demonstrate the impact of the status-neutral approach on targeted testing effectiveness.
2. **Revised Presentation of Results:**
   - In the abstract (lines 48-51), we have restructured the results section to emphasize the key findings related to the status-neutral approach. Specifically, we now highlight how the status-neutral approach enhances the identification of HIV-positive individuals and improves linkage to post-test services, which are critical components of targeted testing effectiveness.
3. **Incorporation of Unexpected Findings:**
   - While we recognize the importance of reporting unexpected findings, we have ensured that these are presented in a way that supports the main research question. Factors associated with HIV seropositivity are now discussed in the context of how they inform and enhance the understanding of the status-neutral approach’s effectiveness.
4. **Main Text Revisions:**
   - We have also revised the analysis, results, and discussion sections of the main text to align with this focus. The revised sections now clearly articulate the role of the status-neutral approach in improving targeted testing outcomes, supported by relevant data and analysis.

We hope these revisions address your concerns and improve the clarity and relevance of our manuscript. Thank you once again for your valuable feedback.

19. REVIEWER COMMENT**:** Another important thing I have noticed in the abstract is that authors talk of screening as the reference point of comparison between status-neutral and targeted testing (correct me if I am wrong). Still to verify at the methods and results section of the paper to confirm this but I already wonder if this status-neutral testing was a complete package or just the screening. It will be good to know what components status-neutral approach were adopted here.

AUTHOR RESPONSE: Thank you for your insightful questions. We appreciate your feedback and would like to provide the following clarifications:

1. **Reference Point of Comparison:**
   - You are correct that the abstract refers to screening as a reference point for comparing the status-neutral and targeted testing approaches. We have clarified this in the methods section to ensure it is clear that the comparison includes all components of the status-neutral approach, not just the screening process.
2. **Components of the Status-Neutral Approach:**

The status-neutral approach adopted in our study is a comprehensive package that includes several key components:

- HIV Testing: Initiating the pathway to prevention and treatment regardless of the test result.
- Linkage to Care: Immediate linkage to HIV care and treatment for those who test positive, and linkage to prevention services for those who test negative.
- Support Services: Integration of supportive services such as counseling, housing, food, and transportation assistance to address social determinants of health.
- Culturally Affirming Care: Providing high-quality, stigma-free, and inclusive care that meets the needs of individuals regardless of their HIV status.

3. **Clarifications in the Manuscript:**
   - We have revised the methods and results sections to explicitly outline these components and how they were implemented in the study. This ensures that readers can clearly understand the full scope of the status-neutral approach used.

We hope these clarifications address your concerns and improve the clarity of our manuscript. Thank you once again for your valuable feedback.

20. REVIEWER COMMENT**:** In clonclusion what overway status-neutral testing to other tasting strategy, Is more effective than PICT, VCT,index testing, Social Network-Based HIV Testing and others strategies

AUTHOR RESPONSE**:** Thank you for your insightful comment. We appreciate the opportunity to clarify the comparative effectiveness of the status-neutral approach relative to other HIV testing strategies.

1. **Effectiveness of Status-Neutral Testing:**

The status-neutral approach is designed to provide comprehensive care and support regardless of HIV status, which helps to reduce stigma and improve engagement in care. This approach integrates HIV prevention and treatment services, addressing the needs of individuals holistically. By focusing on the whole person, the status-neutral approach can enhance retention in care and improve health outcomes.

2. **Comparison with Other Strategies:**
   - **Provider-Initiated Counseling and Testing (PICT):** PICT is effective in clinical settings but This approach integrates HIV prevention and treatment services, addressing the needs of individuals holistically. By focusing on the whole person, the status-neutral approach can enhance retention in care and improve health outcomes may not reach individuals who do not frequently access healthcare services.
   - **Voluntary Counseling and Testing (VCT):** VCT relies on individuals seeking out testing services, which can limit its reach. The status-neutral approach proactively engages individuals and links them to care and prevention services regardless of their test results.
   - **Index Testing:** While index testing is effective in identifying HIV-positive individuals within social networks, it may not address the broader social determinants of health.The status-neutral approach incorporates comprehensive support services, such as housing and mental health care, which can improve overall health outcomes
3. **Social Network-Based HIV Testing:** This strategy effectively reaches high-risk populations but may not provide the same level of integrated care and support as the status-neutral approach. The status-neutral approach ensures that all individuals receive appropriate care and services, regardless of their HIV status

**Conclusion:**

The status-neutral approach offers a more holistic and inclusive strategy by integrating prevention, testing, and treatment services, and addressing social determinants of health. This comprehensive approach can lead to better health outcomes and greater engagement in care compared to other testing strategies.

We hope this response clarifies the advantages of the status-neutral approach and its effectiveness relative to other HIV testing strategies. Thank you once again for your valuable feedback.
