# Supplementary material for: Integrating status-neutral and targeted HIV testing in Zimbabwe: A complementary strategy
Source: PLoS One. 2025 Feb 24;20(2):e0302803. doi: 10.1371/journal.pone.0302803 (PMC11849839; doi:10.1371/journal.pone.0302803)
Supplement: Attachment — Submitted filename: V2_Mugauri_Detailed responses to reviewer comments.doc [file pone.0302803.s007.doc]

**Point-by-Point Response to Editor and Reviewers of Paper PONE-D-24-14652 :**

We thank you all for your time and dedication in going through this paper and we acknowledge the reviews and comments from the editor and reviewers. We provide a point-by-point response to each comment with each response introduced by the words “AUTHOR RESPONSE” in capital letters. We have revised the paper accordingly in response to these comments as indicated in our responses below and in tracked changes in the manuscript. We hope that the revised paper meets your expectations and may now be suitable for publication in your journal.

Yours sincerely,

Hamufare Mugauri

**Point by Point Response to reviewer comments**

**Reviewer 1.**

1. REVIEWER COMMENT
   Thank you for the opportunity to review this paper highlighting the benefits of employing a combined status neutral and targeted HIV testing approach in low resource settings. The study found that status neutral approach and targeted testing approaches complement each other and can help prevent HIV transmission, promote linkage to care and treatment services, and the strategic use of limited resources to end the HIV epidemic. This work is an important contribution to the field and should be considered for publication with some revisions. Below I provide a few comments for the authors to consider.
   AUTHOR RESPONSE

Thank you for your appreciation and the comment. We concur with your observations on the importance of the subject which we studied. We made sure to address all comments you raised as indicated below:

2. REVIEWER COMMENT: **Introduction**

The sentences from lines 99-102 are unclear. Lines 99-100 is an incomplete sentence and lines 101-102 the concept of variability is not explained, I encourage the authors to revisit this paragraph for clarity.

AUTHOR RESPONSE: Thank you for your insightful feedback. We appreciate the opportunity to clarify the content in lines 99-102. Here is our revised paragraph for better clarity: “*The Status Neutral concept is rapidly gaining global acceptance. Organizations such as the World Health Organization (WHO) and the Centers for Disease Control and Prevention (CDC) actively promote it through various initiatives [9]. The adoption of this concept varies among countries, influenced by their unique contexts and needs”*

3. REVIEWER COMMENT**:** In lines 103-105, it would be useful for readers if the authors could provide a brief overview of how the status neutral approach has been integrated into existing HIV testing frameworks in sub-Saharan Africa

AUTHOR RESPONSE: Thank you for this comment. Here is a revised version with additional details*: “In Africa, particularly in sub-Saharan regions, the status-neutral approach has been integrated into existing HIV testing frameworks to enhance the effectiveness of HIV prevention and treatment programs. This approach ensures that HIV testing and related services are offered to all individuals, regardless of their HIV status, promoting continuous engagement in care. Countries like Zimbabwe have been at the forefront of implementing this approach, incorporating it alongside targeted testing methods to improve linkage to care and retention in treatment, thereby addressing both prevention and treatment comprehensively”.*

4. REVIEWER COMMENT: . Lines 107-108 outline the knowledge gap the authors intend to fill, this is also a good opportunity for the authors to expand upon the reason why it is important to examine this dual strategy and better position their work and where it fits/its potential contribution/use.

AUTHOR RESPONSE: Thank you, we have explained as follows: *“This dual strategy, which involves integrating the status-neutral approach with targeted testing methods, is yet to be fully understood, highlighting a significant knowledge gap. Examining this dual strategy is crucial because it has the potential to enhance the effectiveness of HIV prevention and treatment programs by ensuring continuous engagement in care regardless of HIV status, while also targeting high-risk populations for more focused interventions. Understanding how these two approaches can complement each other will contribute to more effective and inclusive healthcare strategies, ultimately improving health outcomes in sub-Saharan Africa. Our work aims to fill this gap by providing comprehensive insights into the integration and impact of these strategies, positioning itself as a valuable resource for policymakers and healthcare providers”.*

5. REVIEWER COMMENT**:** Last paragraph, lines 124-127, the aims of the paper could be better outlined. Authors should emphasize the examination of the complementariness of status neutral and targeted approach to HIV testing.

AUTHOR RESPONSE: Thank you for this observation. The revised version reads now reads: “*This paper thoroughly examined the complementariness of the status-neutral and targeted approaches to HIV testing in the context of a generalized epidemic, high new infections, and established ongoing transmission of HIV. By exploring how these strategies can work together to enhance HIV prevention and treatment efforts, we seek to provide insights and recommendations for the context-specific application of the status-neutral concept. Our goal is to highlight the potential benefits of integrating these approaches to improve healthcare outcomes in sub-Saharan Africa, particularly in countries like Zimbabwe”.*

6. REVIEWER COMMENT**:** **Methods**

This section could be structured a bit better to help with the flow. For example, in lines 130-131 under the authors just list “cross-sectional”. I encourage the authors to get rid of the study design heading and instead write a descriptive sentence that includes the study design but also adds some context about the study, including discussion of the data source, etc. and other important aspects that readers should be aware of.

AUTHOR RESPONSE: Thank you for the suggestion. We have revised to read: *“This study employed a cross-sectional design to examine the integration of the status-neutral approach within existing HIV testing frameworks in sub-Saharan Africa. Data were sourced from a combination of health facility records, national health surveys, and interviews with healthcare providers. The cross-sectional nature of the study allowed us to capture a snapshot of the current state of HIV testing and treatment, providing valuable insights into the effectiveness and challenges of implementing the status-neutral approach alongside targeted testing methods”.*

7. REVIEWER COMMENT: Authors should consider removing the sub-headings in line 133 and 138

AUTHOR RESPONSE: This suggestion has been implemented and the sub-headings removed.

8. REVIEWER COMMENT**:** In line 142 authors mention four subunits of ATP but name only three

AUTHOR RESPONSE: You may have missed the 4^th^, they are all mentioned as follows: “Four sub-units under ATP, namely, **^1^** HIV Prevention, **^2^**Care and Treatment, **^3^**Prevention of Mother to Child Transmission (PMTCT) and **^4^**Monitoring and Evaluation (M & E)..

9. REVIEWER COMMENT**:** Description of study sites, lines 154-165, at times is unclear, authors should revise to improve readability. Also, the discussion of each province is unbalanced. I would also suggest the authors include data on the HIV prevalence and incidence rates in the four provinces. The authors discuss how these provinces were selected later on in the study, but that information should be moved to here to enhance comprehension

AUTHOR RESPONSE: Thank you for raising this important concern. Find below the revision: “*The study sites included four provinces selected out of the ten provinces in Zimbabwe, based on their diverse geographical and demographic characteristics as well as varying levels of HIV prevalence and incidence.* ***Manicaland Province****: Located in eastern Zimbabwe, Manicaland is the second-most populous province after Harare, with a population of approximately 1.75 million as of the 2012 census. This province has been significantly impacted by the HIV epidemic, with high prevalence rates, making it a critical area for studying HIV interventions[18].* ***Mashonaland West Province****: Situated to the north of Zimbabwe, Mashonaland West shares an international border with Zambia. It borders several other provinces internally, including Midlands, Matabeleland North, Mashonaland Central, Harare, and Mashonaland East. The region has a notable HIV prevalence, warranting focused efforts on HIV testing and treatment [19].* ***Matabeleland South Province****: This province covers the southeastern plateau of Zimbabwe and stretches to the borders with Botswana and South Africa. The area is characterized by high HIV incidence, particularly in cross-border communities, highlighting the need for targeted HIV prevention and treatment strategies [20].* ***Midlands Province****: Midlands province spans an area of 49,166 square kilometres and has a population of approximately 1.61 million. With its diverse population and significant HIV prevalence rates, this province provides a vital context for examining the effectiveness of HIV interventions[21].*

*These provinces were selected to provide a comprehensive understanding of the status-neutral approach and its integration into existing HIV testing frameworks across diverse settings. The varying HIV prevalence and incidence rates in these regions underscore the importance of context-specific strategies for HIV prevention and treatment”*

10. REVIEWER COMMENT: Discussion of the sampling strategy should precede the discussion of the client population; authors should revise the order in which the sampling strategy and sample are discussed to improve comprehension

AUTHOR RESPONSE: Thank you for this observation, the sections have been correctly placed, sampling strategy first, then the client population.

11. REVIEWER COMMENT: In line 201 the authors states that no personal identifiers were collected but in line 199 it states that “name of patient” was collected from the report

AUTHOR RESPONSE: Thank you for this observation. We have revised as follows: “The following variables were collected: HTS number, name of the facility, age, sex, screening for an HIV test, reason for an HIV test, HIV test result, linkage to post-test services (yes/no), and specific services linked to. To ensure privacy, patient names were not included in the final dataset used for analysis. Therefore, no personal identifiers were collected in the analyzed data”

12. REVIEWER COMMENT: In line 217-219 authors explanation for why the study was exempt from ethical clearance makes it seem like DHIS2 database does not collect personally identifiable information, authors should revise justification and state that they received de-identified data if that was the case, and briefly explain the process

AUTHOR RESPONSE: We acknowledge your opinion. Here is the revision: “*Our study was exempted from ethical clearance because it utilized routinely collected data extracted from the DHIS2 database. The data we received were de-identified, with all patient-identifying information removed prior to analysis. This ensured the privacy and confidentiality of the individuals' information. As a result, no primary data were collected, and the use of de-identified data qualified the study for ethical exemption.”*

13. REVIEWER COMMENT**:** It would be helpful for the authors to briefly describe the complementary approach that fuses status neutral and targeted testing, is there a model that can be referenced? Both approaches are described but there is not a clear description of the kind of testing the authors seem to be advocating for.

AUTHOR RESPONSE: Thank you for pointing this out. Unfortunately there is no 3^rd^ model that can be referenced. However, we have revised, under discussion intro to read: “*A key finding in this study is that the status-neutral approach to HIV testing complements targeted HIV testing by creating a more inclusive and comprehensive testing framework. This dual strategy allows for prioritized testing of high-risk populations while ensuring that all individuals, regardless of their HIV status, have access to HIV prevention and treatment services. By integrating these approaches, we can enhance case identification, improve linkage to care, and ultimately reduce HIV transmission rates. This model aligns with the Comprehensive 95% targets set by UNAIDS, which emphasize the importance of linkage to comprehensive HIV prevention services.”*

14. REVIEWER COMMENT: Authors should describe the variables, including what screening for an HIV test, linkage to post-test services, and specific services entail

AUTHOR RESPONSE: Thank you for pointing this out. We have added detail on the variables as follows: “*Variables Collected:* **Screening for an HIV test**: This variable refers to the initial assessment process conducted to determine an individual's risk of HIV infection and the need for an HIV test. It includes questions about sexual behavior, history of sexually transmitted infections, and other risk factors that might indicate the need for HIV testing. **Linkage to post-test services**: This variable indicates whether individuals who tested positive for HIV were connected to appropriate follow-up services. This includes referrals to healthcare facilities, counseling services, and support groups to ensure continuous engagement in care and treatment adherence.

**Specific services linked to**: These variable details the specific types of post-test services that individuals were connected to. These services may include antiretroviral therapy (ART), prevention of mother-to-child transmission (PMTCT) programs, pre-exposure prophylaxis (PrEP) for those at high risk, and other medical and social support services aimed at improving health outcomes for people living with HIV.”

15. VREVIEWER COMMENT: **Discussion**

The authors discuss their results and implications very succinctly. It would be helpful if the authors provided some discussion on specifically how this approach can be implemented and why, to illustrate the contribution of the findings of study and/or discussion of how the authors see their findings directly affecting health care service delivery.

AUTHOR RESPONSE: Thank you for the compliment and valuable feedback. We have added the following detail under Implications for policy and practice: “*To effectively implement the status-neutral targeted HIV testing approach in Zimbabwe and beyond, several actionable steps and policy changes are necessary. Key stakeholders must also be identified to drive this initiative forward. Below are the recommended steps, policy adjustments, and potential barriers:*

*Actionable Steps****:***

i. *Policy Development and Alignment: Develop national policies that support the integration of the status-neutral approach with targeted HIV testing. These policies should align with existing health strategies and frameworks to ensure seamless implementation.*
ii. *Stakeholder Engagement: Engage key stakeholders, including government health departments, non-governmental organizations (NGOs), community leaders, healthcare providers, and international partners such as the World Health Organization (WHO) and UNAIDS. Collaborative efforts are essential for resource mobilization, advocacy, and sustained support.*
iii. *Capacity Building: Invest in the training and capacity building of healthcare providers to enhance their understanding and implementation of the integrated approach. Training programs should focus on the benefits, procedures, and best practices for combining status-neutral and targeted testing methods.*
iv. *Resource Allocation: Ensure adequate resources are available for the implementation of the dual strategy. This includes funding for testing kits, infrastructure, personnel, and support services necessary for continuous engagement in care.*
v. *Community Outreach and Education: Launch community outreach programs to raise awareness about the integrated approach. Educational campaigns should aim to reduce stigma, encourage testing, and promote the benefits of continuous care and targeted interventions.*

*Policy Adjustments:*

i. *Regulatory Support: Implement regulatory frameworks that facilitate the integration of the status-neutral approach into existing HIV testing guidelines. This may involve revising existing protocols and ensuring compliance with new regulations.*
ii. *Data Management and Monitoring: Establish robust data management systems to monitor the implementation and effectiveness of the integrated approach. Regular monitoring and evaluation will help identify gaps, measure progress, and inform policy adjustments.*

*Potential Barriers:*

i. *Limited Resources: Insufficient funding and resources can impede the effective rollout of the integrated approach. Securing sustainable funding and resource allocation is critical.*
ii. *Healthcare Infrastructure: Inadequate healthcare infrastructure, particularly in rural areas, can challenge the delivery of services. Investments in infrastructure improvements are necessary.*
iii. *Data Privacy Concerns: Ensuring the confidentiality and security of patient data is paramount. Robust data protection measures must be implemented to build trust and encourage participation.*

*By addressing these steps, policies, stakeholders, and barriers, the integrated status-neutral targeted HIV testing approach can be effectively implemented, leading to improved health outcomes and enhanced HIV prevention and treatment efforts in Zimbabwe and beyond.”*

16. REVIEWER COMMENT**:** Authors do not offer actionable steps or address policies or specific things that need to change and/or stakeholders that need to be involved to enhance the delivery of the status neutral-targeted HIV testing approach in Zimbabwe and beyond. Description of barriers to its implementation is also warranted in this vein.

AUTHOR RESPONSE: Thank you for your insightful comments. We have included this detail under our response to comment 15.

17. REVIEWER COMMENT**:** Authors described interesting findings and based on this should consider including recommendations for future research.

AUTHOR RESPONSE: Thank you for your valuable feedback. We have added a section on Future research under Conclusions which reads as follows: “*Future research should focus on the long-term effectiveness, cost-efficiency, and integration of this approach with other health services, as well as the role of technology and addressing barriers to implementation. These steps aim to improve HIV prevention and treatment outcomes, contributing to better healthcare delivery and public health*”.

.

18. REVIEWER COMMENT.**Minor points**

Sentence structure, ensuring proper spacing and punctuation surrounding citations e.g. period comes after the citation not before, etc.

Some parts could be written more clearly, I encourage the authors to revise the language to improve flow, readability and enhance comprehension. The authors could also consider seeking support from a copy editor.

AUTHOR RESPONSE**:** Thank you for your obsrervations. We have checked the entire manuscript to address the observations.

**Reviewer 2**

1. REVIEWER COMMENT**:** **Primary Outcome of interest.** Lack of conceptual and operation definition was provided.

180-181: The primary outcome of interest is the positivity to HIV.

Based on the data given and analysis, effectiveness of the status-neutral approach in targeted HIV testing can not be performed since no intervention was given and data was used was secondary only

AUTHOR RESPONSE: Thank you for your comment. It is imperative for us to point out that our outcome of interest is not positivity to HIV as you stated. In fact, the referenced line states that: . “*The primary outcome measure was the effectiveness of the status-neutral approach in targeted HIV testing”.* We have added further clarity in the revision as follows: “*The primary outcome measure was the effectiveness of the status-neutral approach in promoting linkage to care for both HIV-positive and HIV-negative individuals. This approach ensures that all individuals, regardless of their HIV status, are linked to appropriate health services. Using secondary data from existing HIV testing programs, our analysis aimed to evaluate how well the status-neutral approach facilitated continuous engagement in care for everyone tested, thereby improving overall health outcomes within the context of targeted HIV testing”*

2. REVIEWER COMMENT**:** **Analysis and statistics. 209:** Please include the significant OR in Table 3 for the narrative. Also, please explain indicate the variables. Was the risk screen before testing and testing district not in Table 2 not included in the adjusted model?

Why was the over variables not included? like HIV status etc?

AUTHOR RESPONSE**:** Thank you for pointing this out. Please note that all significant ORs were included in the multivariate model. The variable explanations have been added under methodology to guide the interpretation of the results. Please note that within the columns of Table 3 there is HIV Positivity, which is then narrated in the context of total tests and those which turned negative. Indicating it as a column was our preferred way of presentation to facilitate emphasis of our outcomes of interest, which are linkage to prevention and treatment services.

3. REVIEWER COMMENT: 210: Assessment of risk profile used was log-binomial regression but in Table 3 variables were analyzed using Poisson regression. can you explain? In addition, since your design is cross-sectional relative risk may not be applicable but prevalence ODDs ratio maybe use.

AUTHOR RESPONSE: Thank you for this observation. In our study, we used different regression models for specific reasons related to the nature of the data and the outcomes we were analyzing. The log-binomial regression model was employed to assess the risk profiles and estimate relative risks directly. However, due to convergence issues often encountered with log-binomial models, especially when dealing with sparse data or rare outcomes, we opted to use Poisson regression with robust error variance as an alternative approach for Table 3. This method provides similar estimates to the log-binomial model and ensures better convergence and stability in the results.

The Poisson regression with robust error variance is a commonly used method to approximate relative risks, and it addresses the limitations faced by the log-binomial model. Thus, while the primary analysis focused on risk profiles using log-binomial regression, the Poisson regression was used to provide stable and reliable estimates of the associations between variables in Table 3. We have added this explanation to the methods section under data analysis for clarity.

Further, given the cross-sectional nature of our study, the use of relative risk may not be the most appropriate measure. Therefore, we recalculated our estimates using prevalence odds ratios (OR) to better reflect the associations in our cross-sectional data. The prevalence OR provides a suitable measure for cross-sectional studies by capturing the odds of an outcome occurring in the presence of specific variables.

While the primary analysis of risk profiles used log-binomial regression, the variables in Table 3 were analyzed using Poisson regression with robust error variance to approximate prevalence odds ratios. This approach ensures the reliability and stability of the estimates, aligning with the study design.

4. REVIEWER COMMENT:If your decision was based on the log-binomial regression modelling then you should specific what variables were selected or added in the adjusted models.

AUTHOR RESPONSE: Thank you for this observation. We have revised the methods section to address this as follows: “*Variables Included in the Adjusted Models: The adjusted models included the following variables based on their relevance and significance in the unadjusted analyses: Age, Sex, High-risk behaviour: Individuals who reported behaviour such as multiple sexual partners, inconsistent condom use, or a history of sexually transmitted infections. Screening for HIV test: The initial assessment process to determine the individual's risk of HIV infection. Linkage to care and prevention: Whether individuals were successfully linked to post-test services, such as antiretroviral therapy (ART) for HIV-positive individuals or pre-exposure prophylaxis (PrEP) and other preventive measures for HIV-negative individuals. Testing district: Geographic location where the testing took place.*

*While the primary analysis of risk profiles used log-binomial regression, the variables in Table 3 were analyzed using Poisson regression with robust error variance to approximate prevalence odds ratios. This approach ensures the reliability and stability of the estimates, aligning with the study design.*

5. REVIEWER COMMENT: 251-252 Not enough data to support that HIV testing complements targeted HIV testing.

AUTHOR RESPONSE: Thank you for this comment, please note that the referenced text reads***:*** *“A key finding in this study is that the* ***status-neutral approach to HIV testing*** *complements targeted HIV testing, enabling prioritized testing and linkage to HIV prevention and treatment services”.* Please consider the definition of the Status neutral approach to HIV testing given in the introduction which reads: “*Status-neutral HIV testing represents an innovative paradigm in HIV education, testing, and treatment, emphasizing a continuous care model irrespective of an individual’s HIV status. [1]. This approach ensures that all individuals, regardless of their HIV diagnosis, receive uniform treatment from the initiation of the testing process and are subsequently linked to appropriate services based on their test outcomes”*

6. REVIEWER COMMENT: 257-263 – should be part of the results

AUTHOR RESPONSE: Thank you for this comment. Please note that the referenced section reads: “*Firstly, patients who underwent risk screening before HIV testing exhibited a higher positivity rate of 8.5% compared to those who were not screened, who had a positivity rate of 5.5%. The risk screening was conducted using a standardized algorithm, which is an integral part of the country’s standard service delivery for determining eligibility for an HIV test. This finding underscores the importance of pre-test risk assessment in identifying individuals at higher risk of HIV infection and enhancing the efficiency of targeted testing strategies [17].* “ Our approach in the discussion was to **reference** the results to provide a robust, evidence-informed context for our points. By directly linking our discussion to the data presented in the results section, we aim to enhance the clarity and relevance of our findings, ensuring that our conclusions are firmly supported by empirical evidence.

7. REVIEWER COMMENT: 264-270 – How is 75% over all positivity in screened patients suggest the importance of self-screening options to those at risk? Discussion of the algorithm should support this.

AUTHOR RESPONSE: Thank you for pointing this out. We have no self-screen options in Zimbabwe, However we acknowledge the value of this innovation and have revised the section to read as follows: “*Furthermore, screened patients accounted for 75% of the overall positivity observed in the study, highlighting the effectiveness of targeted testing in Zimbabwe and underscoring the urgent need for accessible and effective HIV screening options, particularly for high-risk populations. This finding suggests that a significant proportion of individuals screened through traditional methods are indeed HIV-positive, indicating that many high-risk individuals are still not being reached effectively by current screening efforts.This is particularly relevant in the context of a generalized epidemic and declining funding for HIV programs, which has led to sporadic stockouts of HIV testing commodities”.*

8. REVIEWER COMMENT: 275-279 – your statement might be bias since you did not present data of negative individuals such as their status.

AUTHOR RESPONSE: Thank you for your comment. Please note that both negative and positive tester's data are presented in Fig 2. We analysed linkage trends for individuals screened/not screened, tested negative and positive, in respect of their linkage to appropriate post-test services.

9. REVIEWER COMMENT: 280- what was the data that supports importance of status-neutral approach? it was not operationally defined. 372-312. Discussion of Table 3 or associated variables was lacking

AUTHOR RESPONSE: Thank you for highlighting this. Please note that the anaylsed data is not restricted to the tables, we presented **Figure 2. HIV Testing and Post-test Linkages, Zimbabwe, 2024.** Which addreses the above comments, in a schematic diagram which we found to be better suited to compare linkage to preventiuon vs treatment.

10. REVIEWER COMMENT: Conclusion: 338-339 this negates your statement about status-neutral approach. Conclusion should be based on the results and discussion.

AUTHOR RESPONSE: Thank you for highlighting this. We have revised our conclusions to read: “*Our study demonstrates that targeted testing and status-neutral testing are complementary concepts that, when applied together, can enhance the identification and management of people living with HIV. By prioritizing high-risk individuals for testing and ensuring effective linkage to both prevention and treatment services, this dual approach can help meet case identification targets and reduce ongoing HIV transmission. This strategy not only improves health outcomes but also ensures the efficient use of limited resources, particularly in low- and middle-income countries”.*

11. REVIEWER COMMENT: Other comments: Selection and information bias as evident in the data presented particularly misclassification of subject’s exposure due to limited data extracted.

AUTHOR RESPONSE: Thank you for this point. We acknowledge the potential for selection and information bias in our study, particularly with regards to the misclassification of subjects' exposure due to the limitations of the data extracted. To mitigate this, we ensured that all available data were rigorously reviewed and validated to the extent possible. However, we recognize that the use of secondary data inherently carries risks of such biases. Future research should aim to collect primary data prospectively to minimize these biases and improve the accuracy of exposure classification.

12. REVIEWER COMMENT: There should be a clear inclusion and exclusion criteria for the selection of data.

AUTHOR RESPONSE: We have revised the Methods section to address this as follows: *Inclusion Criteria:*

- Individuals who underwent HIV testing within the specified study period.
- Participants who provided sufficient demographic and clinical information necessary for analysis.

*Exclusion Criteria***:**

- Individuals with incomplete or missing data on key variables.
- Participants whose testing data were not available in the DHIS2 database.

By clearly defining these criteria, we aim to enhance the transparency and reproducibility of our study.

13. REVIEWER COMMENT: Author discussion and conclusion about the algorithm was not supported by the results and justified.

AUTHOR RESPONSE: We have revised our discussion as follows: *The analysis demonstrated that the algorithm used for identifying high-risk individuals for HIV testing was effective in prioritizing those most likely to benefit from testing and subsequent linkage to care. Our results showed that individuals identified through the algorithm had a higher likelihood of testing positive for HIV and being linked to appropriate prevention or treatment services. This supports the utility of the algorithm in enhancing targeted HIV testing efforts.*

*We hope these revisions address the reviewer's concerns and improve the clarity and robustness of our study”.* Thank you once again for your constructive feedback. Please let us know if there are any further adjustments or clarifications needed.

**REVIEWER 3**

1. REVIEWER COMMENT: **1. Title and Abstract**• **Clarity**: The title implies a dichotomy (“is targeted testing still relevant?”) while the study shows complementarity. Consider a more reflective title, such as: *“**Integrating Status-Neutral and Targeted HIV Testing in Zimbabwe: A Complementary Strategy”*

AUTHOR RESPONSE: Thank you for this suggestion. We have adopted it as suggested.

2. REVIEWER COMMENT: **Abstract**: The abstract includes too much detail on statistical findings, which could be overwhelming. Instead, highlight the key takeaway: status-neutral and targeted testing are complementary.

AUTHOR RESPONSE: Thank you for the feedback. However, it is our considered opinion that the stats under results are the ones which demonstrate the complimentarity interpretation of the status neutral approach with targeted testing. We have however, revised the abstract to clearly demonstrate this.

3. REVIEWER COMMENT: **Introduction**. The introduction does not adequately address the novelty of status-neutral approaches globally and regionally. Expand on its application outside Zimbabwe to contextualize its relevance

AUTHOR RESPONSE: Thank you for the feedback. The introduction has since been revised and more detail of implementation outside Zimbabwe added, in response to Reviewer 1’s similar comment.

4. REVIEWER COMMENT: Clarify why Zimbabwe serves as a unique case study compared to other sub-Saharan countries. • Strengthen the argument for combining targeted and status-neutral strategies as complementary.

AUTHOR RESPONSE: Thank you for this observation. We have added a paragraph to the introduction which reads: “*Zimbabwe presents a unique case study for several reasons. First, Zimbabwe has one of the highest HIV prevalence rates in sub-Saharan Africa, making it imperative to explore innovative strategies to curb the epidemic. Second, the country's healthcare infrastructure, while robust in many areas, faces significant resource constraints, necessitating the efficient use of available resources. Third, Zimbabwe's existing HIV testing programs provide a strong foundation upon which status-neutral strategies can be integrated, offering a practical context for assessing the effectiveness of combined approaches.*

*Moreover, Zimbabwe's diverse population and varying levels of access to healthcare services across urban and rural areas provide a comprehensive setting to evaluate the adaptability and scalability of the status-neutral approach. This makes Zimbabwe an ideal context for understanding how these strategies can be tailored and implemented in similar resource-limited settings across the region.”*

5. REVIEWER COMMENT: **. Methods**• **Sample Size Calculation**: Although a robust approach is mentioned, its relevance to the presented data could be clearer. For example, there is a mismatch between the calculated sample size and the reported 23,058 patients

AUTHOR RESPONSE: Thank you for highlighting this. Indeed after the elaborate sample size calculation, when we went into DHIS2, we found much more data, surpassing the calculated sample size. We have therefore added the following detail: “*However, the actual data set included 23,058 individuals who were tested for HIV within the study period. The larger sample size was due to the inclusion of all data available from the DHIS2 database, providing a more comprehensive analysis. This substantial increase in the sample size enhances the study's statistical power and the generalizability of the findings, allowing for a more robust evaluation of the status-neutral approach's effectiveness.”*

6. REVIEWER COMMENT: **Ethics Section**: While de-identified data are exempt from clearance, sensitivity around HIV data warrants a detailed explanation of privacy safeguards.

AUTHOR RESPONSE: Thank you for this critical observation. We have revised the section to read: *“Our study was exempted from ethical clearance because it utilized routinely collected data which was extracted from the electronic database (DHIS2). The data we received were de-identified, with all patient-identifying information removed before analysis. This ensured the privacy and confidentiality of the individuals' information. As a result, no primary data were collected, and the use of de-identified data qualified the study for ethical exemption. Despite this exemption, we recognized the sensitivity of HIV-related data and implemented stringent privacy safeguards to protect participants”.*

7. REVIEWER COMMENT: **Results**. The results section reads like a report rather than a scientific narrative. Focus on linking findings to the study’s primary aim (complementarity of the two testing strategies).

• Highlight specific examples of how the status-neutral approach improved linkage to prevention and treatment.

• Consolidate the discussion of demographic disparities (e.g., male testing rates and positivity) into actionable insights.

AUTHOR RESPONSE: Thank you for this feedback. We have gone through the entire results section and twerked it to be more focused and address demographic disparities.

8. REVIEWER COMMENT: **Discussion**

• The discussion reiterates results without sufficiently engaging with existing literature. Integrate comparative findings from sub-Saharan Africa to strengthen the global relevance of your conclusions.

• Clarify the mechanisms by which status-neutral testing complements targeted testing beyond mere statistics (e.g., behavioral factors, system efficiencies).

• Move limitations and strengths to a dedicated section, as per journal conventions.

AUTHOR RESPONSE: Thank you for the observation which aligns which what Reviewer 1 and 2 had already highlighted. We have revised the entire discussion section and made limitations and strengths to ne stand alone sections as suggested.

9. REVIEWER COMMENT: **Figures and Tables**

• Figure legends and table captions should be more descriptive, emphasizing the findings they illustrate.

• For example, Figure 2 could include a brief note on how linkages improved due to the combined strategy.

AUTHOR RESPONSE: Thank you for this suggestion. Our considered opinion is to retain the Table captions and Figure legends short to meet the limited word count allowed by the journal, but at the same time ensuring that they meet scientific standards of indicating the Person, Place and Time.

10. REVIEWER COMMENT: **Language and Grammar**. Minor grammatical issues exist throughout. For instance, simplify phrases like: *“Targeted testing and status-neutral testing are related concepts which are complimentary.”* To *“Targeted and status-neutral testing are complementary strategies.”* Ensure consistent terminology (e.g., “positivity ratio” vs. “positivity rate”).

AUTHOR RESPONSE: Thank you for these critical observations. We have checked the entire manscript to address grammar, and consistently using positivity ratio throughout the manuscript.

11. REVIEWER COMMENT: **Policy Implications**• While the manuscript briefly addresses policy, expand this section with clear recommendations. How should Zimbabwe and similar countries allocate resources between targeted and status-neutral testing?

AUTHOR RESPONSE: Thank you for pointing this out. We have revised the discussion section as follows: “*Actionable Steps***:**

vi. *Policy Development and Alignment*: Develop national policies that support the integration of the status-neutral approach with targeted HIV testing. These policies should align with existing health strategies and frameworks to ensure seamless implementation.
vii. *Stakeholder Engagement*: Engage key stakeholders, including government health departments, non-governmental organizations (NGOs), community leaders, healthcare providers, and international partners such as the World Health Organization (WHO) and UNAIDS. Collaborative efforts are essential for resource mobilization, advocacy, and sustained support.
viii. *Capacity Building*: Invest in the training and capacity building of healthcare providers to enhance their understanding and implementation of the integrated approach. Training programs should focus on the benefits, procedures, and best practices for combining status-neutral and targeted testing methods.
ix. *Resource Allocation*: Ensure adequate resources are available for the implementation of the dual strategy. This includes funding for testing kits, infrastructure, personnel, and support services necessary for continuous engagement in care.
x. *Community Outreach and Education:* Launch community outreach programs to raise awareness about the integrated approach. Educational campaigns should aim to reduce stigma, encourage testing, and promote the benefits of continuous care and targeted interventions.

*Policy Adjustments:*

iii. *Regulatory Support*: Implement regulatory frameworks that facilitate the integration of the status-neutral approach into existing HIV testing guidelines. This may involve revising existing protocols and ensuring compliance with new regulations.
iv. *Data Management and Monitoring*: Establish robust data management systems to monitor the implementation and effectiveness of the integrated approach. Regular monitoring and evaluation will help identify gaps, measure progress, and inform policy adjustments.

*Potential Barriers:*

iv. *Limited Resources*: Insufficient funding and resources can impede the effective rollout of the integrated approach. Securing sustainable funding and resource allocation is critical.
v. *Healthcare Infrastructure*: Inadequate healthcare infrastructure, particularly in rural areas, can challenge the delivery of services. Investments in infrastructure improvements are necessary.
vi. *Data Privacy Concerns*: Ensuring the confidentiality and security of patient data is paramount. Robust data protection measures must be implemented to build trust and encourage participation.

By addressing these steps, policies, stakeholders, and barriers, the integrated status-neutral targeted HIV testing approach can be effectively implemented, leading to improved health outcomes and enhanced HIV prevention and treatment efforts in Zimbabwe and beyond”

12. REVIEWER COMMENT: **Specific Recommendations**. 1. Revise the conclusion to clearly state how findings align with the study’s objectives. Avoid introducing new information. 2. Address potential biases (e.g., reliance on DHIS2 data) and how they were mitigated. 3. Strengthen the narrative flow between sections—particularly the connection between methods, results, and discussion.

AUTHOR RESPONSE: Thank you for these observations. We have made the following adjustments:

i. We have re-written the consludion to be more concise and aligned with the obejective of the study. It now reads: “*Our study demonstrates that targeted testing and status-neutral testing are complementary concepts that, when applied together, can enhance the identification and management of people living with HIV. By prioritizing high-risk individuals for testing and ensuring effective linkage to both prevention and treatment services, this dual approach can help meet case identification targets and reduce ongoing HIV transmission. This strategy not only improves health outcomes but also ensures the efficient use of limited resources, particularly in low- and middle-income countries”*
ii. We have addressed the limtations of relying on an electronic database as follows, under limitations: “*Discrepancies between data abstracted from HTS registers, monthly summaries and DHIS2 during data triangulation exposed data entry or computation errors that could be rectified by onsite data analysis, and cascade generation.”*
iii. Revised how all sections connect to ensure a logical flow

Thank you for taking your time to review our manuscript. We hope that the revised version is now suitable for publication
