# Supplementary material for: Integrating status-neutral and targeted HIV testing in Zimbabwe: A complementary strategy
Source: PLoS One. 2025 Feb 24;20(2):e0302803. doi: 10.1371/journal.pone.0302803 (PMC11849839; doi:10.1371/journal.pone.0302803)
Supplement: Attachment — Submitted filename: V3_Mugauri_Detailed responses to reviewer comments.doc [file pone.0302803.s010.doc]

**Point-by-Point Response to Editor and Reviewers of Paper PONE-D-24-14652 :**

We thank you all for your time and dedication in going through this paper once more and we acknowledge the reviews and comments from the editor and reviewers. We provide a point-by-point response to each comment with each response introduced by the words “AUTHOR RESPONSE” in capital letters. We have revised the paper accordingly in response to these comments as indicated in our responses below and in tracked changes in the manuscript. We hope that the revised paper meets your expectations and may now be suitable for publication in your journal.

Yours sincerely,

Hamufare Mugauri

REVIEWER COMMENT: Thank you for providing the opportunity to re-review the paper post revisions from the authors. I am satisfied with all the changes. One minor suggestion I have is that, in addition to the detail provided on the included provinces, I recommend that the authors to consider including the prevalence rates for each province into the manuscript.

AUTHOR RESPONSE

Thank you for your appreciation and the comment. . We are pleased to hear that you are satisfied with the changes we have made. We appreciate your suggestion to include the prevalence rates for each province. We agree that this addition would enhance the depth and clarity of our manuscript. We have therefore incorporated the prevalence rates for Manicaland, Mashonaland West, Matabeleland South, and Midlands Province in the revised version of the paper as follows: “*Manicaland Province: Located in eastern Zimbabwe, Manicaland is the second-most populous province after Harare, with a population of approximately 1.75 million as of the 2022 census. This province has been significantly impacted by the HIV epidemic, with a prevalence of 9.40% in 2024. . This makes it a critical area for studying HIV interventions[18, 19]. Mashonaland West Province: Situated to the north of Zimbabwe, Mashonaland West shares an international border with Zambia. It borders several other provinces internally, including Midlands, Matabeleland North, Mashonaland Central, Harare, and Mashonaland East. The region had a notable HIV prevalence of 9.60% in 2024, warranting focused efforts on HIV testing and treatment [19, 20]. Matabeleland South Province: This province covers the southeastern plateau of Zimbabwe and stretches to the borders with Botswana and South Africa. The area is characterized by high HIV incidence, particularly in cross-border communities, with a prevalence rate of 17.30% in 2024, highlighting the need for targeted HIV prevention and treatment strategies [19, 21]. Midlands Province: Midlands province spans an area of 49,166 square kilometres and has a population of approximately 1.61 million. With its diverse population and significant HIV prevalence rate of 10.94% in 2024, , this province provides a vital context for examining the effectiveness of HIV interventions[19, 22].”*
